# *Typha* for paludiculture—Suitable water table and nutrient conditions for potential biomass utilization explored in mesocosm gradient experiments

**DOI:** 10.1002/ece3.9191

**Published:** 2022-08-23

**Authors:** Kerstin Haldan, Nora Köhn, Anja Hornig, Sabine Wichmann, Jürgen Kreyling

**Affiliations:** ^1^ Institute of Botany and Landscape Ecology University of Greifswald, partner in the Greifswald Mire Centre Greifswald Germany

**Keywords:** gradient experiment, paludiculture, productivity, tissue chemical composition, *Typha*, utilization

## Abstract

Drainage has turned 650,000 km^2^ of peatlands worldwide into greenhouse gas sources. To counteract climate change, large‐scale rewetting is necessary while agricultural use of rewetted areas, termed paludiculture, is still possible. However, more information is required on the performance of suitable species, such as cattail, in the range of environmental conditions after rewetting. We investigated productivity and biomass quality (morphological traits and tissue chemical composition) of *Typha angustifolia* and *Typha latifolia* along gradients of water table depth (−45 to +40 cm) and nutrient addition (3.6–400 kg N ha^−1^ a^−1^) in a six‐month mesocosm experiment with an emphasis on their high‐value utilization, e.g., as building material, paper, or biodegradable packaging. Over a wide range of investigated conditions, *T. latifolia* was more productive than *T. angustifolia*. Productivity was remarkably tolerant of low nutrient addition, suggesting that long‐term productive paludiculture is possible. Low water tables were beneficial for *T. latifolia* productivity and high water tables for *T. angustifolia* biomass quality. Rewetting will likely create a mosaic of different water table depths. Our findings that the yield of *T. angustifolia* and tissue chemical composition of *T. latifolia* were largely unaffected by water table depth are therefore promising. Depending on intended utilization, optimal cultivation conditions and preferable species differ. Considering yield or diameter, e.g., for building materials, *T. latifolia* is generally preferable over *T. angustifolia*. A low N, P, K content, high Si content and high C/N‐ratio can be beneficial for processing into disposable tableware, charcoal, or building material. For these utilizations, *T. angustifolia* is preferable at high water tables, and both species should be cultivated at a low nutrient supply. When cellulose and lignin contents are relevant, e.g., for paper and biodegradable packaging, *T. angustifolia* is preferable at high water tables and both species should be cultivated at nutrient additions of about 20 kg N ha^−1^ a^−1^.

## Graphical abstract

In a six‐month mesocosm experiment, we investigated productivity and biomass quality (morphological traits, tissue chemical composition) of promising paludiculture crops *Typha angustifolia* and *Typha latifolia* along gradients of water table depth and nutrient supply with an emphasis on the plants' high‐value utilization (e.g. as building material, paper, biodegradable packaging). Biomass quality requirements, optimal cultivation conditions and preferred species differ depending on intended utilization: Within the scope of investigated conditions, *T. latifolia* was generally more productive than *T. angustifolia*, productivity was remarkably tolerant of low nutrient supply and these conditions benefitted tissue chemical composition. *T. angustifolia* productivity and *T. latifolia* tissue chemical composition remained stable across water tables, however, low water tables were beneficial for *T. latifolia* productivity, high water tables beneficial for *T. angustifolia* biomass quality.

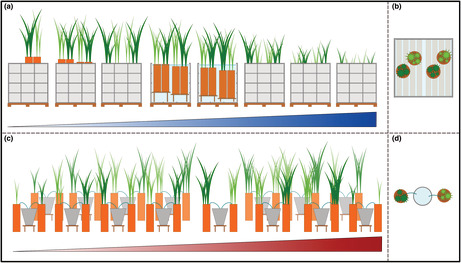

## 1 INTRODUCTION

Peatlands are one of the most effective terrestrial ecosystems to sequester carbon (C) as organic matter (Knox et al., 2015; Rocha & Goulden, 2009). Globally, 650,000 km^2^ of peatlands have been drained, mainly for agriculture, and thereby turned into carbon sources (Joosten & Clarke, 2002). Therefore, peatland restoration is a very effective measure for greenhouse gas (GHG) emission mitigation (Leifeld & Menichetti, 2018). To substantially reduce net GHG emissions from the peatland biome, all drained peatlands need to be rewetted and large‐scale rewetting should consequently start now (Günther et al., 2020; Leifeld et al., 2019; Tanneberger et al., 2021). Conventional drainage‐based agriculture is no longer possible on rewetted peatlands. To enable a sustainable use of the vast areas, alternative practices such as their wet use, termed paludiculture (Wichtmann et al., 2016a), have to be explored. However, the response of target crops to the variety of environmental conditions after rewetting, especially to higher nutrient loads and stronger water table fluctuations than under natural peatland conditions (Kreyling et al., 2021), are not yet investigated comprehensively.

Suitable target crop species for paludiculture are adapted to wet conditions and their biomass utilization is economically viable (Abel et al., 2013). *Typha* spp. fulfill these requirements: they can grow under waterlogged and even flooded conditions and produce more dry matter (>20 t dry matter ha^−1^ a^−1^ reported for North America and South Germany (Dubbe et al., 1988; Pfadenhauer & Wild, 2001) or about 10–15 t dm ha^−1^ a^−1^ on rewetted semi‐natural sites in our study region in Midwest Europe (Geurts et al., 2020; Zerbe et al., 2013)) than conventional intensive grassland use on the same sites while drained (11–14 t dm ha^−1^ a^−1^; Geurts & Fritz, 2018).

N‐availability for economically viable *Typha* paludiculture is suggested to be 100 kg N ha^−1^ a^−1^ (Geurts & Fritz, 2018), which is lower than the input from fertilization and net N mineralization under grassland use in drained conditions (up to 340 kg N ha^−1^ a^−1^ from fertilization and up to 600 kg N ha^−1^ a^−1^ from net N mineralization; Schrautzer, 2004; Tiemeyer et al., 2016). Thereby paludiculture can reduce the risk of eutrophication of downstream water bodies. *Typha* paludiculture furthermore offers a large range of biomass utilization options.

*Typha* biomass is well suited as a building material mainly for construction and insulation as about 85% of its tissue consist of aerenchyma, giving the material good insulation properties, a low density, and simultaneously high break resistance and bearing capacity (Georgiev et al., 2014; Krus et al., 2014). Climate benefits associated with the use as building material are emission reduction during cultivation, substitution of fossil raw materials, and long‐term carbon storage in the products (Lahtinen et al., 2022; Pittau et al., 2018). For the use as building material plants must, however, fulfill certain requirements including, but not limited to their morphological properties, such as height and diameter. While a low water content is desirable (Wichtmann et al., 2016b), *Typha* plants keep a high water content of up to 32%–60% at winter harvest (Maddison, Soosaar, et al., 2009) and biomass has to be predried for storage and most applications (Geurts et al., 2020; Geurts & Fritz, 2018). Paper and biodegradable packaging are other promising high‐value utilizations (Alebiosu et al., 2019; Reich, 2019). For these materials, high cellulose and low lignin contents are required and plant fiber dimensions may be of importance (Ververis et al., 2004). However, the biomass quality requirements for different utilizations remain unknown and vague in many cases, the reasons being that *Typha* biomass is rarely used as raw material or utilizations are still in a test phase.

In field cultivation, many factors can influence the productivity and biomass quality of crops, including species, genotype, water table, nutrient supply, and harvest date (Geurts et al., 2020; Kasak et al., 2020; Maddison, Mauring, et al., 2009; McNaughton, 1974; Pijlman et al., 2019). Especially water tables and nutrient supply can be difficult to control in large‐scale paludiculture, implying that farmers will need to optimize their crop selection and its usage under the given conditions. Yet, research directly comparing *Typha* species and the quantity and quality of their biomass production under various environmental conditions is rare. Previous studies with *Typha* often include only one species (Li et al., 2004; Ren et al., 2019; Wetzel & van der Valk, 1998) or focus on either nutrient or water supply (Deegan et al., 2007; Hong et al., 2020; Macek & Rejmánková, 2007).

*Typha angustifolia* (*T. angustifolia*) and *Typha latifolia* (*T. latifolia*) are the most relevant *Typha* species for paludiculture in Germany. They occur over a wide range of nutrient availability (growing at total N additions of 0–1330 kg N ha^−1^ a^−1^ under controlled conditions (Geurts & Fritz, 2018; Ren et al., 2019; Wild et al., 2001)). *T. angustifolia* is often found to be more productive than *T. latifolia* under field and experimental conditions (Dubbe et al., 1988; Grace & Wetzel, 1981). Both species produce more above‐ and belowground biomass (Grace, 1988; Jordan et al., 1990; Macek & Rejmánková, 2007; Ren et al., 2019), develop more shoots (Grace, 1988; Ren et al., 2019), a greater shoot diameter and height (Ren et al., 2019), and greater rhizome length (Macek & Rejmánková, 2007) with increasing nutrient availability. Higher nutrient availability also leads to increased N and P concentrations in plant tissue (Jordan et al., 1990; Miao et al., 2000; Ren et al., 2019).

*Typha* species are highly productive under waterlogged conditions and show decreased productivity when facing prolonged (water table 30 cm below soil surface) or periodic (soil water potential down to −0.8 Mpa) soil moisture deficits (Geurts & Fritz, 2018; Li et al., 2004). *T. latifolia* outcompetes *T. angustifolia* in shallow water (<50 cm) but *T. angustifolia* is better adapted to growing in deeper water depths (Grace & Wetzel, 1981, 1982). While both species develop aerenchyma as an adaption to flooding (Constable et al., 1992; Ni et al., 2014), flooding may lead to light limitation for photosynthesis, which can be counteracted by increased height growth (Grace, 1989; Grace & Wetzel, 1982; Heinz, 2012). The effect of water table on biomass production is not clear: Some studies report no effect of flooding on total biomass (Byun et al., 2017; Hong & Kim, 2016) while others show the highest productivity of young *Typha* plants under water tables at soil surface but reduced biomass production at lower or higher water tables (Heinz, 2012).

To optimize *Typha*‐based paludiculture on rewetted peatlands as a profitable alternative to drainage‐based agriculture, we need to increase our understanding on how *Typha* species perform under a wide range of environmental conditions. Therefore, we conducted a mesocosm study in which we directly compared the performance of *T. angustifolia* and *T. latifolia* along a water table gradient and a nutrient addition gradient in two parallel experiments. We quantified belowground biomass production, which is the basis for peat production, C sequestration, and growth in the following season (McNaughton, 1974; Rocha & Goulden, 2009; Schwieger et al., 2020; Succow & Joosten, 2001), physiological activity (photosynthesis), and the quantity and quality (morphological traits and tissue composition) of aboveground biomass.

We hypothesized for both species that (1a) biomass productivity reaches an optimum at water tables close to soil surface and photosynthetic rate decreases with increasing water tables and (1b) biomass quality for high‐value utilization is optimal at high water tables as this should result in higher physical and chemical resistance to physical disturbance (water/wave movement) and rotting (low N, P, K content, high Si content). We furthermore expected (2a) biomass productivity and photosynthetic rate to increase with increasing nutrient addition and (2b) biomass quality to generally decrease with increasing nutrient addition (high N, P, K content) with notable exceptions such as stem diameter and potentially also Si content being positively influenced by increasing nutrient addition. With regards to each of the two species, we hypothesized that (3a) *T. latifolia* reaches its optimum performance at lower water tables than *T. angustifolia* as the latter was expected to have its optimum performance at higher water tables, that (3b) the two species reach optimal performance at a similar nutrient addition and that (3c) *T. angustifolia* generally outperforms *T. latifolia*.

Finally, we discuss the implications of our results for the use of *Typha* spp. in paludiculture of rewetted temperate fen ecosystems.

## 2 MATERIAL AND METHODS

We grew two species of cattail, *Typha angustifolia* and *Typha latifolia*, in a mesocosm experiment and assessed their performance along gradients of (A) nutrient addition and (B) water table height.

### 2.1 Experimental setup

We collected seeds of both *Typha* species in February 2019 in a naturally established stand near Lake Kummerow in Mecklenburg‐Western Pomerania. In April 2019, seeds were sown on trays with an equal mixture of sand, loamy soil, and commercial substrate (Naturtalent by toom® Bio‐Universalerde, toom Baumarkt GmbH, Köln, Germany). Trays were kept in a greenhouse at 22°C and soil was kept moist at all times using tap water. Trays were rotated regularly to ensure equal conditions for all plants. In May 2019, plants were potted into multi‐pot plates using bog peat as substrate (*Sphagnum* peat, limed, not fertilized, pH 5.6–6.4; Torfwerk Moorkultur Ramsloh, Germany; element content in % dry matter (DM): 0.95% DM total N (Kjeldahl method), 0.026% DM total P (Aqua regia digestion, ICP‐OES), 0.06% DM K (Aqua regia digestion, ICP‐OES), 0.15% DM Mg (Aqua regia digestion, ICP‐OES), 0.13% Fe (Aqua regia digestion, ICP‐MS)) and transferred outside.

The mesocosm experiment was conducted from June to November 2019 in the Arboretum at the University of Greifswald, Germany. The experiment focused on the detection of potentially nonlinear patterns along environmental gradients. Gradient experiments maximizing treatment levels by minimizing replication per treatment level are powerful tools for this kind of question (Kreyling et al., 2018). Consequently, we set up one replicate per species per treatment level, consisting of three plants that were planted in one plastic tube on 11^th^ June 2019. Plants were grown in plastic tubes (height 60 cm, diameter 20 cm) filled with the same bog peat substrate. Communal tap water (0.014 mg L^−1^ ${\mathrm{NH}}_{4}^{+}$, 0.016 mg L^−1^ ${\mathrm{NO}}_{2}^{-}$, 1.72 mg L^−1^ ${\mathrm{NO}}_{3}^{2-}$, 0.038 mg L^−1^ ${\mathrm{PO}}_{4}^{3-}$, 2.84 mg L^−1^ K^+^ [calculated from data of Stadtwerke Greifswald GmbH, n.d.]) was used for irrigation, employing a gravity‐feed system: Water was filled into an elevated rain barrel and from there distributed to water reservoirs for each nutrient treatment and containers for water table treatments.

**FIGURE 1:**
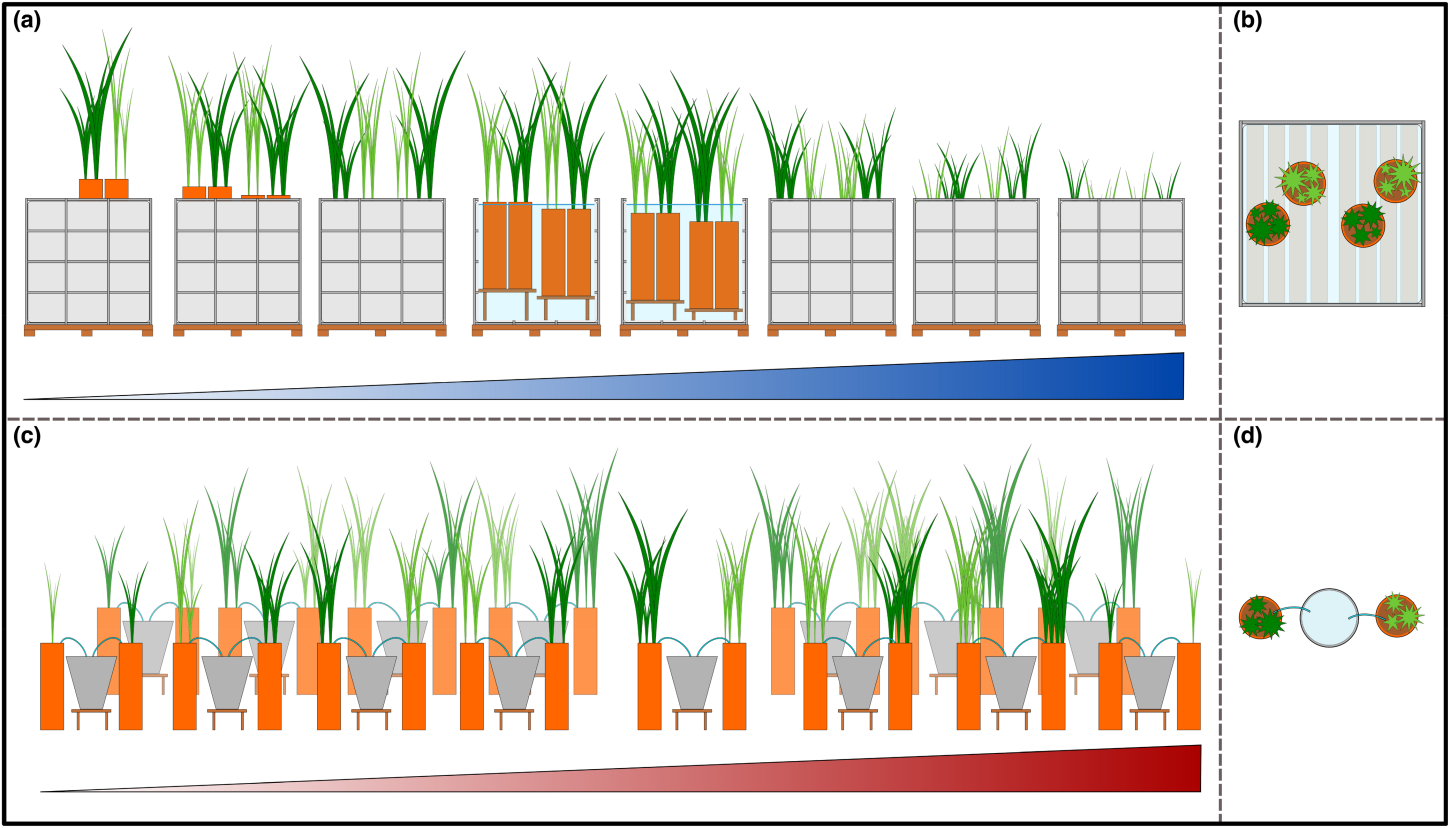
Schematic setup of the mesocosm experiment, visualizing the underlying gradients. In the water table gradient ((a) side view, (b) top view), tubes (orange) with *Typha angustifolia* (light green), and *Typha latifolia* (dark green) were placed on platforms of different heights inside 1000 L containers. Fifteen levels formed a water table gradient ranging from water table 45 cm below soil surface to 40 cm above soil surface. In the nutrient addition gradient ((c) side view, (d) top view), tubes with *T. angustifolia* and *T. latifolia* of each level were connected to their own water reservoir (gray buckets). Fifteen levels formed a gradient of nutrient addition ranging from the addition of 3.6–400 kg N ha^−1^ a^−1^. In reality, gradient levels were arranged randomly.

For the water table treatments, plants were grown in a gradient of 15 water tables ranging from a water table 45 cm below soil surface to 40 cm above soil surface (Table 1). Tubes were closed at the bottom with two layers of water permeable root fleece (polypropylene, 150 g m^−2^). Tubes were placed on wooden platforms inside 1000 L water containers (1 × 1 × 1 m) to achieve the desired water tables. In each container, two different water levels were simulated by constructing wooden platforms of different heights (Figure 1). While all water tables below soil surface were kept constant from the beginning of the experiment, water tables above soil surface were raised according to plant growth until the intended water table was reached, which was the case for all levels at the beginning of August. To provide the plants with the macronutrients (N, P, and K), they were fertilized with a nutrient solution amounting to 37.9 kg N ha^−1^ a^−1^ equivalent to the middle level (Level 8) of the nutrient addition treatment, which was prepared in the way described below. The water table treatments were fertilized bi‐weekly starting on 11^th^ June 2019. At the first fertilization, a third of the yearly nutrient dose was given, and the remaining amount of nutrients was split over the remaining six dates. 0.5 L of nutrient solution was poured directly into each tube (water level at or below soil surface) or in the water above each tube (water levels above soil surface).

**TABLE 1:** Water table in relation to soil surface in the water table gradient. Positive numbers are water tables above soil surface, negative numbers are water tables below soil surface

Level	Water table [cm]
1	−45
2	−38
3	−31
4	−24
5	−18
6	−12
7	−6
8	0
9	+5
10	+10
11	+16
12	+22
13	+28
14	+34
15	+40

For the nutrient treatments, plants were grown in a nutrient addition gradient, ranging from addition of 3.6–400 kg N ha^−1^ a^−1^ in a nutrient solution, not taking into account additional nutrient sources such as the substrate, water, or atmospheric deposition. The gradient consisted of 15 levels with increasing nitrogen (N) addition by a factor of 1.4 (Table 2). In addition to nitrogen, phosphorus (P) and potassium (K) were supplied. We chose an N/P‐ratio of 10 as previous studies by Tylová et al. (2013) and Romero et al. (1999) found this ratio to be suitable for the growth of emergent wetland plants. We chose an N/K‐ratio of 1.452, according to measurements of pore water in fens across central to eastern Europe (Tanneberger, 2019). Tubes were sealed at the bottom, and the water table was kept at soil surface constantly. Each treatment had a separate water reservoir to avoid mixing of nutrient concentrations among treatments (Figure 1). For the nutrient addition, the target amount of nitrogen was scaled down to the surface area of a tube (314.16 cm^2^) and then split over 7 dates at intervals of 2 weeks throughout the growing season. At the first fertilization (11^th^ June 2019), a third of the yearly nutrient dose was given, and the remaining amount of nutrients was split over the remaining six dates. The nutrient solution was prepared by dissolving NH_4_NO_3_, (NH_4_)_2_HPO_4_, and K_2_CO_3_ in demineralised water (Table 2). For a nutrient addition treatment, the water table in the tubes was lowered to allow the nutrient solution to penetrate the soil. Then, 0.5 L of nutrient solution was poured into each tube. After the first nutrient addition, some *Typha* plants in the higher nutrient addition treatments died. All dead plants were replaced by healthy plants on 21^st^ June 2019. To avoid this happening again, we split the nutrient addition of the highest treatments over 2 days in the further course of the experiment.

**TABLE 2:** Amount of fertilizer used in the fertilization gradient: Target amount of nitrogen (N) [kg ha^−1^ a^−1^] and resulting amount of chemicals (NH4)2HPO4, NH4NO3, and K_2_CO_3_ used for fertilization per pot [g a^−1^] based on a pot surface area of 314.16 cm^2^, an N/P‐ratio of 10, and an N/K‐ratio of 1.452.

Level	Target amount of N [kg ha^−1^ a^−1^]	Total amount of fertilizer per pot [g a^−1^]		
		(NH_4_)_2_HPO_4_	NH_4_NO_3_	K_2_CO_3_
1	3.6	0.131	0.371	0.545
2	5.0	0.184	0.519	0.763
3	7.1	0.257	0.727	1.069
4	9.9	0.360	1.018	1.496
5	13.8	0.504	1.425	2.094
6	19.4	0.706	1.995	2.932
7	27.1	0.988	2.793	4.105
8	37.9	1.383	3.910	5.747
9	53.1	1.936	5.474	8.046
10	74.4	2.710	7.664	11.264
11	104.1	3.795	10.729	15.770
12	145.8	5.312	15.021	22.078
13	204.1	7.437	21.029	30.909
14	285.7	10.412	29.441	43.273
15	400.0	14.577	41.218	60.582

The mesocosms experienced ambient weather conditions without any kind of roof. During the experimental period from May 2019 to November 2019, the lowest minimum temperature measured was −2°C (1^st^ November 2019) and the highest maximum temperature was 37°C (30^th^ June 2019). The coldest month was November 2019 with a mean minimum temperature of 4.4°C and mean maximum temperature of 7.6°C while the warmest month was June 2019 with a mean minimum temperature of 15.5°C and a mean maximum temperature of 25.6°C. Monthly precipitation ranged from 29 mm in July 2019 to 96 mm in November 2019.

### 2.2 Data collection

We measured the number of leaves on the highest shoot on each plant in mid‐September at the end of the growing season. Plants were harvested at the end of November 2019. At harvest, height and maximum diameter of 10 randomly chosen shoots and total shoot number per tube were measured. Fresh and dry (48 h, 80°C) weight of aboveground biomass (per tube) were assessed and content of lignin, cellulose, total C, total N, P, K, and Silicon (Si) at LUFA Rostock, Germany.

Except for C (DIN ISO 10694), N, P, K and Si were analyzed following the procedures described in the Association of German Agricultural Analytic and Research Institute's book of methods (Verband Deutscher Landwirtschaftlicher Untersuchungs‐ und Forschungsanstalten, 1976, chapter 4.1.2 (N), 10.8.3 (P, K, Si)). For lignin and cellulose, an extended Weender analysis according to van Soest was conducted, in which acid detergent fiber after combustion (ADF om) and acid detergent lignin (ADL, hereafter referred to as “lignin”) were determined. Cellulose content was calculated from ADL and ADF om according to Formula 1 by subtracting ADL from ADF om (Verband Deutscher Landwirtschaftlicher Untersuchungs‐ und Forschungsanstalten, 1976, chapter 6.5.2–6.5.3).

(1) $$\text{Cellulose content}\;\left[g\;{\mathrm{kg}}^{-1}\;\mathrm{DM}\right]=\mathrm{ADF}\;\mathrm{om}\;\left[g\;{\mathrm{kg}}^{-1}\;\mathrm{DM}\right]-\mathrm{ADL}\;\left[g\;{\mathrm{kg}}^{-1}\;\mathrm{DM}\right]$$

Water content of aboveground biomass was calculated from its fresh and dry weight according to Formula 2.

(2) $$\text{water content}\;\left[\%\right]=\frac{\left(\text{fresh weight}\;\left[g\right]-\mathrm{dry}\;\text{weight}\;\left[g\right]\right)}{\text{fresh weight}\;\left[g\right]}\times 100$$

C/N‐ratio was calculated as a mass‐based C/N‐ratio.

Belowground biomass was washed, separated into roots and rhizomes (per tube), and dried at 60°C until constant dry weight. We recorded the dry weight of roots and rhizomes at 0.01 g precision.

Photosynthetic rate was quantified by leaf gas exchange using the LCi‐T Accessible Photosynthesis System (ADC BioScienfitic Limited, Hoddesdon, UK) with the external RGB LED light unit (constant PAR [Qleaf]: 1430 μmol m^−2^ s^−1^ with equal parts of red, green and blue light) on the first measurement date (5^th^ August 2019) and the LCi‐SD Leaf Chamber Analysis System (ADC BioScienfitic Limited) with the external PLU5 LED light unit (given PAR, PAR Q_leaf_ (g) = 1481 μmol m^−2^ s^−1^, PAR (Q) = 1600 μmol m^−2^ s^−1^) on the following two dates (22^nd^/23^nd^ August 2019 and 18^th^/19^th^ September 2019), always fitted with the narrow leaf chamber without radiation shield (area: 5.80 cm^2^, Hfac: 0.168, rb: 0.30, Tl_mtd_: measured, Trw = 0.92). At each measurement date, the photosynthetic rate was measured on three plants per species per treatment at the second fully expanded leaf of each plant. To avoid a possible influence of noon depression on photosynthesis, the three measurements per species per treatment were distributed over the course of the day and rotated between measurement dates. A reading was taken when values of gas exchange and photosynthetic rate of a leaf in the gas exchange chamber had stabilized after about 2 to 4 min. If a reading was taken at a leaf too narrow to fit the chamber, the measured photosynthetic rate ($A_{\text{orig}}$) was corrected for leaf width filling the chamber (leaf width [cm]) according to Formula 3 to give a corrected photosynthetic rate ($A_{\text{corr}}$).

(3) $$A_{\text{corr}}=A_{\text{orig}}*\frac{1}{\text{leaf width}\;\left[\mathrm{cm}\right]}$$

When it was not possible to measure on the second fully expanded leaf from the top and another leaf was chosen, photosynthetic rate $A_{\text{corr}}$ was corrected for leaf position on the stem. For this purpose, the photosynthetic rate of all leaves of a plant was additionally measured on three plants in different nutrient levels and four plants in different water tables per species. Percentage photosynthetic rate at each leaf position in relation to leaf position two (%dev) was calculated and photosynthetic rate ($A_{\text{corr}}$) was corrected to resemble the photosynthetic rate at leaf position two ($A_{\text{leaf}}$) according to Formula 4:

(4) $$A_{\text{leaf}}=\frac{A_{\text{corr}}*100}{\%\mathrm{dev}}$$

From these values, the mean photosynthetic rate per level and species was calculated.

### 2.3 Statistical analysis

This study was designed as a gradient experiment (Kreyling et al., 2018), aiming to observe patterns of response variables over gradients of the environmental drivers nutrient availability and water table. To unravel these nonlinear response patterns, a graphical analysis was performed in R (Version 3.6.0; R Core Team, 2019) using the package ggplot2 (Version 3.3.0; Wickham, 2016). Each response parameter was plotted over the environmental gradient and smoothed conditional means were calculated by Local Polynomial Regression Fitting using the “loess” function implemented in R. Span was adjusted to produce smooth curves without multiple extrema. This was done based on the assumption that several minima and maxima along the gradients would not be ecologically plausible in such an autecological setting without competition. Response values reported in the results were obtained from the Local Polynomial Regression Fitting. Confidence intervals (CIs) displayed around the conditional means were used to assess significant effects at a level of α = 0.05. The effect of an environmental driver on the response parameter was considered significant if a straight horizontal line could not be fitted inside the 95% CI (Gelman & Hill, 2007). The species were considered significantly different if their 83% CIs did not overlap as the comparison of two 83% CIs approximate an alpha level of 5% (Austin & Hux, 2002; Knol et al., 2011; Payton et al., 2003).

### 2.4 Practitioner survey

In April and May 2021, an international survey was conducted to collect information from practitioners about biomass requirements for various utilization options. Prior to the survey, companies already using paludiculture biomass or other plant biomass as raw material for their products were identified. A questionnaire was designed with questions regarding currently used raw materials, quality requirements for raw materials, manufactured products, willingness to substitute other plant‐based raw materials with paludiculture biomass (e.g., *Typha* spp., *Phragmites australis*, wet meadow grasses), limiting factors to use paludiculture biomass, required volume and processing state, and price expectations. In this publication, we focused on statements on quality requirements and refrained from evaluating the other topics.

## 3 RESULTS

### 3.1 Water table gradient

**FIGURE 2:**
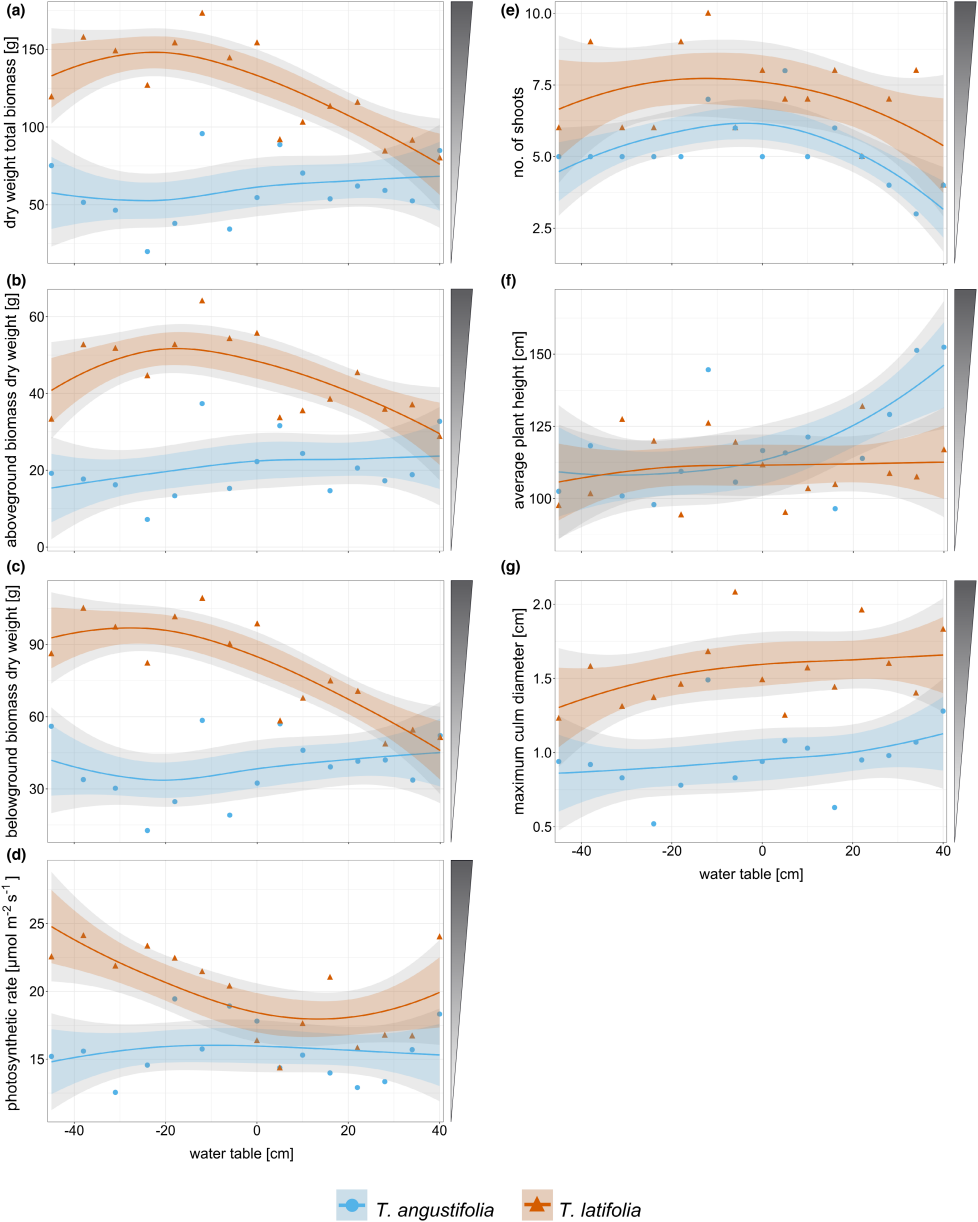
Dry weight [g] of (a) total biomass (span = 1.4), (b) aboveground biomass (span = 1.5), and (c) belowground biomass (span = 1.5); (d) photosynthetic rate [μmol m^−2^ s^−1^] (span = 2.5) and (e) number of shoots (span = 1.4), (f) average shoot height [cm] (span = 2.5), and (g) maximum clum diameter [cm] (span = 1.5) at harvest of *Typha angustifolia* (blue) and *Typha latifolia* (red) along the water table gradient. Negative water tables are below soil surface and positive water tables above soil surface. Dots are original data points, and lines show the smoothed local polynomial regression fitting (loess). 95% confidence intervals are displayed in gray, and 83% confidence intervals are displayed in color. Shaded triangles next to plots indicate preferred (=dark) biomass properties for high‐value utilization.

**FIGURE A1:**
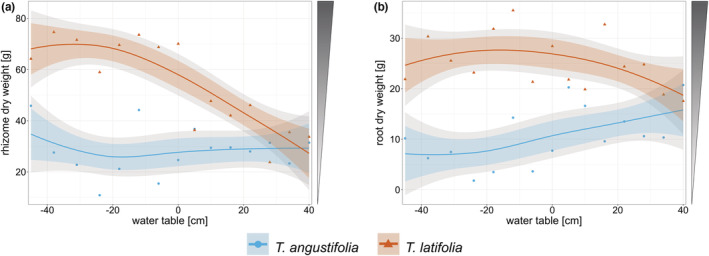
(a) Rhizome (a, span = 1.5) and (b) root (b, span = 1.5) dry weight [g] of *Typha angustifolia* (blue) and *Typha latifolia* (red) along the water table gradient. Negative water tables are below soil surface and positive water tables above soil surface. Dots are original data points, and lines show the smoothed local polynomial regression fitting (loess). 95% confidence intervals are displayed in gray, and 83% confidence intervals are displayed in color. Shaded triangles next to plots indicate preferred (= dark) biomass properties for high‐value utilization.

Total biomass production of *T. angustifolia* was not affected by water table, whereas *T. latifolia* produced more biomass at water tables below and close to soil surface, and its biomass production decreased with increasing flooding (Figure 2a). *T. latifolia* reached a maximum total biomass of 148 g (aboveground 52 g, belowground 97 g) at a water table of 22 cm below ground, whereas *T. angustifolia* reached a maximum total biomass of 68 g (aboveground 24 g, belowground 45 g) at a water table of 40 cm above ground. Above‐ and belowground biomass (Figure 2b,c), and root and rhizome biomass (Figure A1) showed the same patterns along the water table gradient, with the exception that root biomass of *T. latifolia* did not change along the gradient.

Over a large part of the water table gradient (−45 to +30 cm), *T. latifolia* produced significantly and up to 2.8 times more total biomass than *T. angustifolia*. Only at the highest flooding treatments of 30 cm and more, the species did not differ in their biomass production.

**FIGURE A2:**
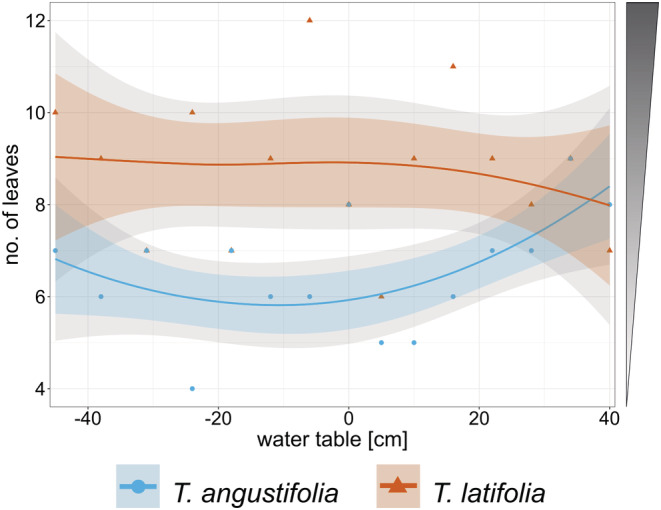
Number of leaves per shoot (span = 2.0) measured on 18^th^ September 2019, at the end of the growing season, of *Typha angustifolia* (blue) and *Typha latifolia* (red) along the water table gradient. Negative water tables are below soil surface and positive water tables above soil surface. Dots are original data points, and lines show the smoothed local polynomial regression fitting (loess, span = 1.5). 95% confidence intervals are displayed in gray, and 83% confidence intervals are displayed in color. The shaded triangle next to the plot indicates preferred (= dark) biomass properties for high‐value utilization.

Shoot number and height showed a different pattern than biomass along the water table gradient at harvest. Water table had no significant effect on number of shoots (5–8, Figure 2e), leaves per shoot (8–9, Figure A2) and average shoot height (106–113 cm, Figure 2f) of *T. latifolia*. *T. angustifolia* on the other hand produced significantly less (max. 6 shoots at −4 cm) but higher shoots (max. shoot height 146 cm at +40 cm) and more leaves per shoot (max. 8 leaves at +40 cm) under flooded conditions. *T. angustifolia* grew significantly taller than *T. latifolia* under flooding (approx. +20 to +40 cm), but *T. latifolia* produced more shoots and more leaves per shoot than *T. angustifolia* over a large part of the water table gradient (approx. −40 to −5 cm and +5 to +35 cm; approx. −40 to +25 cm, respectively). The maximum culm diameter of both species did not change over the water table gradient (Figure 2g). The diameter of *T. latifolia* (1.3–1.7 cm) was significantly and up to 1.7 times higher than the diameter of *T. angustifolia* (0.9–1.1 cm).

**FIGURE 3:**
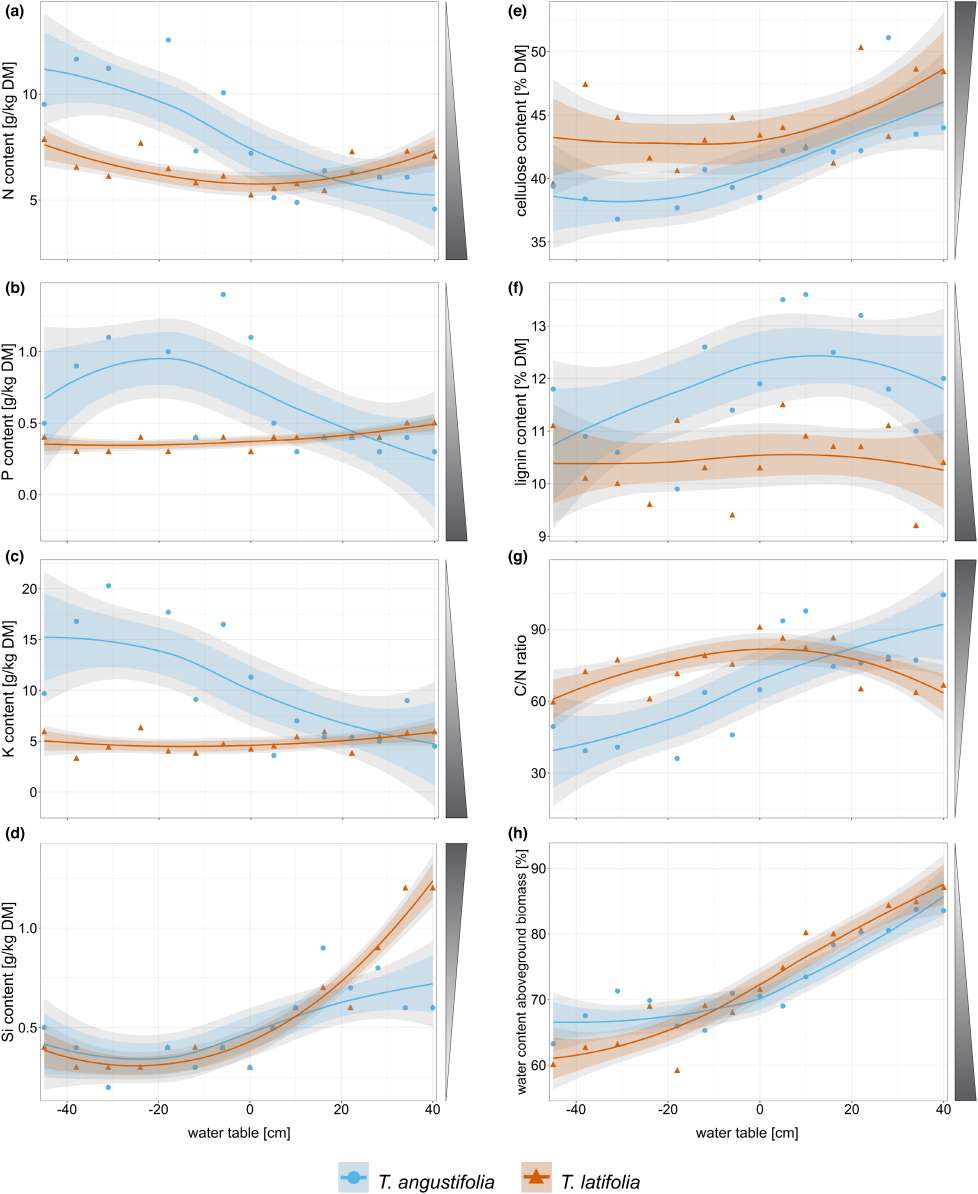
Content [g kg^−1^ dry matter] of (a) N (span = 1.4), (b) P (span = 1.2), (c) K (span = 1.8) and (d) Si (span = 1.5), content [% dry matter] of (e) cellulose (ADF om – ADL, span = 1.5) and (f) lignin (ADL, span = 2.3), (g) mass‐based C/N‐ratio (span = 1.5), and (h) water content [%] (span = 1.1) in aboveground biomass of *Typha angustifolia* (blue) and *Typha latifolia* (red) along the water table gradient. Negative water tables are below soil surface and positive water tables above soil surface. Dots are original data points, and lines show the smoothed local polynomial regression fitting (loess). 95% confidence intervals are displayed in gray, and 83% confidence intervals are displayed in color. Shaded triangles next to plots indicate preferred (=dark) biomass properties for high‐value utilization.

**FIGURE A3:**
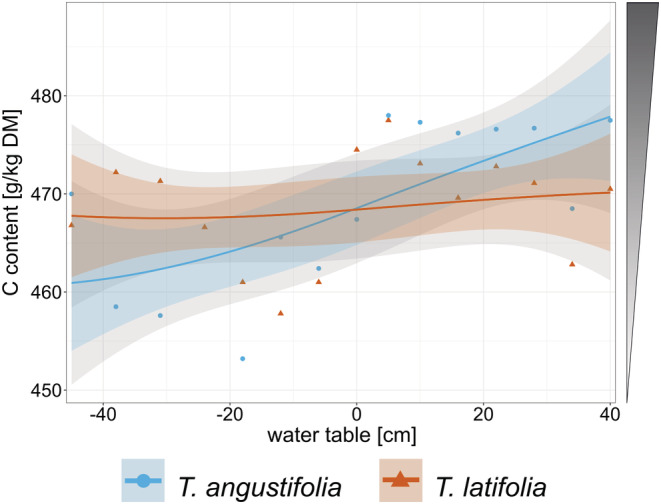
C content [g kg^−1^ dry matter] (span = 3.0) in aboveground biomass of *Typha angustifolia* (blue) and *Typha latifolia* (red) along the water table gradient. Negative water tables are below soil surface and positive water tables above soil surface. Dots are original data points, and lines show the smoothed local polynomial regression fitting (loess, span = 1.5). 95% confidence intervals are displayed in gray, 83% confidence intervals are displayed in color. The shaded triangle next to the plot indicates preferred (= dark) biomass properties for high‐value utilization.

Photosynthetic rate of *T. latifolia* decreased significantly from dry to flooded conditions while photosynthetic rate of *T. angustifolia* did not change over the water table gradient (Figure 2d). *T. latifolia* reached a maximum photosynthetic rate of 24.8 μmol m^−2^ s^−1^, *T. angustifolia* of 16.0 μmolm^−2^ s^−1^. *T. latifolia* showed a significant and up to 1.7 times higher photosynthetic rate than *T. angustifolia* under dry (approx. −5 to −45 cm) and partly under flooded conditions (approx. +22 to +37 cm). Macronutrient content (N, P, K, Figure 3a–c) in the shoots was hardly affected by the water table gradient in *T. latifolia* (N content 5.76–7.62 g kg^−1^, P content 0.35–0.49 g kg^−1^, K content 4.48–5.89 g kg^−1^) while it decreased to half (N, P) or even one third (K) of the maximum nutrient content with increasing water tables in *T. angustifolia* (N content 5.24–11.18 g kg^−1^, P content 0.24–0.49 g kg^−1^, K content 4.27–15.22 g kg^−1^). C content (Figure A3) and C/N‐ratio (Figure 3g) were also less responsive to the water table gradient in *T. latifolia* (C content 467.52–470.13 g kg^−1^, C/N‐ratio 60.82–81.74) than in *T. angustifolia*, where they generally increased with higher water tables (C content 460.93–477.87 g kg^−1^, C/N‐ratio 39.45–92.01). Si concentrations in the shoots were constant for water tables below surface to water table at surface height but doubled (*T. angustifolia*, 0.34–0.72 g kg^−1^) or even tripled (*T. latifolia*, 0.31–1.24 g kg^−1^) when the plants were flooded (Figure 3d).

Water table had no influence on lignin content in either species (Figure 3f) and on cellulose content in *T. latifolia*, cellulose content of *T. angustifolia* did not change at water tables below ground but increased at high water tables (Figure 3e). *T. angustifolia* had a significantly higher lignin content than *T. latifolia* over most of the water table gradient (approx. −30 to +38 cm) but a significantly lower cellulose content than *T. latifolia* at water tables below ground (approx. −5 to −40 cm).

Water content of aboveground biomass increased in both species significantly towards more flooded conditions (Figure 3h). *T. latifolia* showed an increase from 61% to 88% and *T. angustifolia* from 67% to 86%. At water tables below ground (approx. −25 to −42 cm), *T. angustifolia* had a higher water content than *T. latifolia*; however, *T. latifolia* had a higher water content than *T. angustifolia* at water tables above ground (approx. +15 to +25 cm).

### 3.2 Nutrient gradient

**FIGURE 4:**
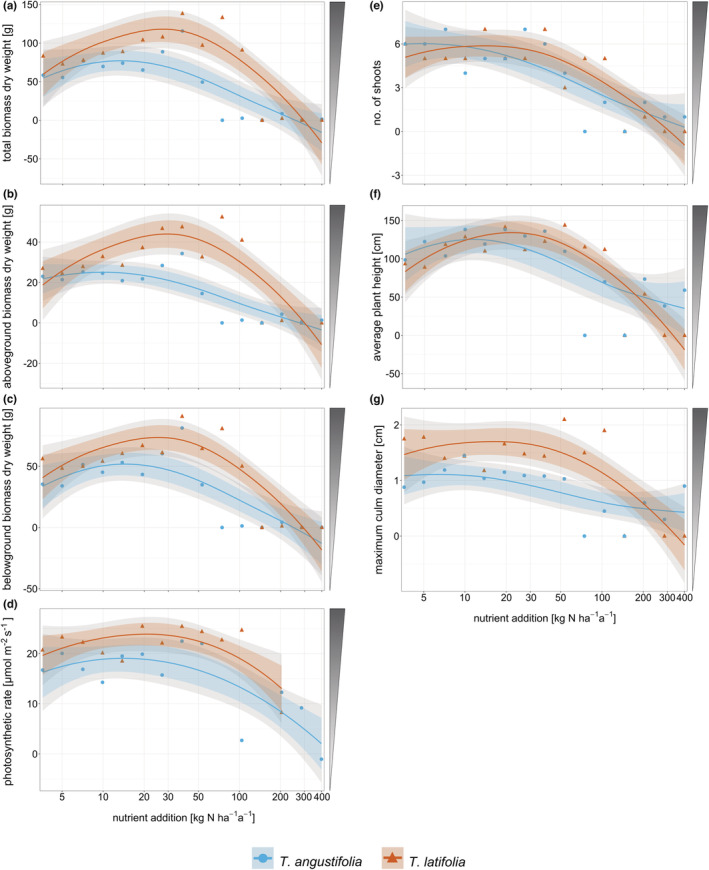
Dry weight [g] of (a) total biomass (span = 1.4), (b) aboveground biomass (span = 1.4), and (c) belowground biomass (span = 1.5); (d) photosynthetic rate [μmol m^−2^ s^−1^] (span = 2.5) and (e) number of shoots (span = 1.4), (f) average shoot height [cm] (span = 1.4) and (g) maximum clum diameter [cm] (span = 2.0) at harvest of *Typha angustifolia* (blue) and *Typha latifolia* (red) along the nutrient addition gradient. Dots are original data points, and lines show the smoothed local polynomial regression fitting (loess). 95% confidence intervals are displayed in gray, and 83% confidence intervals are displayed in color. Shaded triangles next to plots indicate preferred (= dark) biomass properties for high‐value utilization.

**FIGURE A4:**
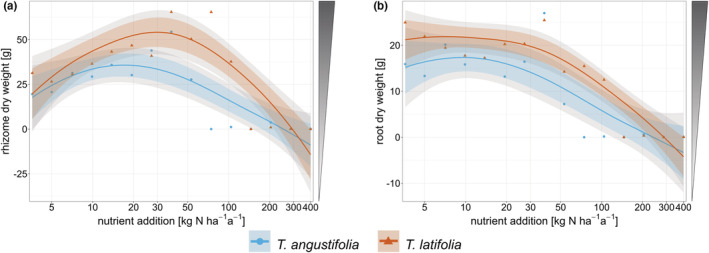
(a) Rhizome (span = 1.4) and (b) root (span = 1.4) dry weight [g] of *Typha angustifolia* (blue) and *Typha latifolia* (red) along the nutrient addition gradient. Dots are original data points, and lines show the smoothed local polynomial regression fitting (loess). 95% confidence intervals are displayed in gray, and 83% confidence intervals are displayed in color. Shaded triangles next to plots indicate preferred (= dark) biomass properties for high‐value utilization.

Total biomass of both *Typha* species did not change significantly at low to intermediate nutrient addition but decreased significantly at high nutrient addition (Figure 4a). The same pattern was observed for above‐ and belowground biomass (Figure 4b,c), and root and rhizome biomass (Figure A4). Only rhizome biomass of *T. latifolia* showed a significant increase from low to intermediate nutrient addition, with an optimum at 29 kg N ha^−1^ a^−1^. *T. angustifolia* reached a maximum total biomass productivity of 77 g (aboveground: 25 g; belowground: 52 g) at a nutrient addition of 13 kg N ha^−1^ a^−1^ while *T. latifolia* reached a maximum total biomass productivity of 118 g (aboveground: 44 g; belowground: 74 g) at a higher nutrient addition of 27 kg N ha^−1^ a^−1^. *T. latifolia* was significantly more, and up to 3.3 times as productive as *T. angustifolia* in a range of approx. 12–180 kg N ha^−1^ a^−1^, but the productivity of the species did not differ significantly at low and very high nutrient additions. The differences in productivity between the species were more pronounced in aboveground biomass compared to belowground biomass.

**FIGURE A5:**
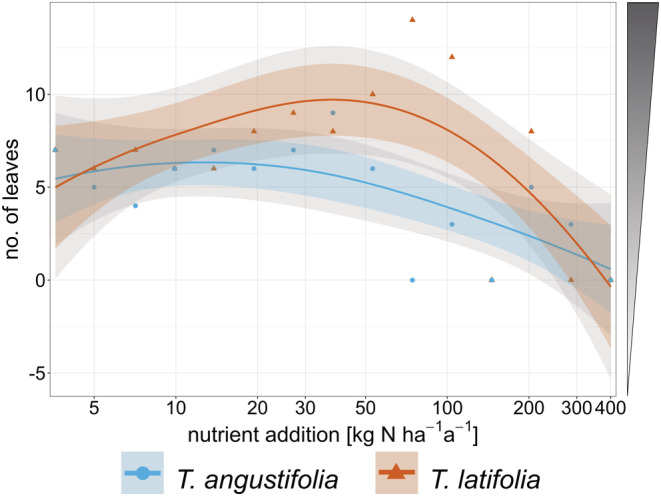
Number of leaves per shoot (span = 1.5) measured on 18^th^ September 2019, at the end of the growing season, of *Typha angustifolia* (blue) and *Typha latifolia* (red) along the nutrient addition gradient. Dots are original data points, and lines show the smoothed local polynomial regression fitting (loess). 95% confidence intervals are displayed in gray, and 83% confidence intervals are displayed in color. The shaded triangle next to the plot indicates preferred (= dark) biomass properties for high‐value utilization.

Average plant height and shoot number at harvest (Figure 4e,f) and leaf number at the end of the growing season (Figure A5) decreased significantly with increasing nutrient addition in *T. angustifolia*. Height and leaf number of *T. latifolia* followed the shape of an optimum curve with a nonsignificant increase at low to intermediate and a significant decrease at high nutrient addition. Shoot number of *T. latifolia* was unaffected at low nutrient addition and decreased significantly from approx. 20 kg N ha^−1^ a^−1^ onwards. The *Typha* species did not differ significantly in shoot number or height, but *T. latifolia* produced more leaves than *T. angustifolia* at intermediate nutrient addition (approx. 25–170 kg N ha^−1^ a^−1^). *T. latifolia* reached its maximum number of shoots (6 at 14 kg N ha^−1^ a^−1^), leaves (10 at 37 kg N ha^−1^ a^−1^), and height (134 cm at 22 kg N ha^−1^ a^−1^) at a higher nutrient addition than *T. angustifolia* (shoots: 6 at 5 kg N ha^−1^ a^−1^, leaves: 6 at 13 kg N ha^−1^ a^−1^, height 125 cm at 11 kg N ha^−1^ a^−1^).

Culm diameter of *T. latifolia* (maximum culm diameter 1.7 cm) decreased significantly at high nutrient addition and was significantly and up to two times higher at a range of approx. 7–160 kg N ha^−1^ a^−1^ than the diameter of *T. angustifolia* (0.4–1.1 cm), which did not change significantly over the whole nutrient gradient (Figure 4g).

The photosynthetic rate of both *Typha* species remained stable over most of the nutrient gradient and only decreased at very high nutrient additions (Figure 4d). At a high nutrient addition of 200 kg N ha^−1^ a^−1^ and more, the photosynthetic rate of *T. latifolia* could not be measured because all plants had died or were too small for measurements. For the same reason, only a few measurements could be made on *T. angustifolia* at very low (3.6 kg N ha^−1^ a^−1^) and high (74.4–400 kg N ha^−1^ a^−1^) levels of nutrient addition.

At a range of nutrient addition from approx. 30–130 kg N ha^−1^ a^−1^, *T. latifolia* showed a significantly higher photosynthetic rate than *T. angustifolia*. *T. latifolia* reached a maximum photosynthetic rate of 24 μmol m^−2^ s^−1^ at 22 kg N ha^−1^ a^−1^, *T. angustifolia* of 19 μmol m^−2^ s^−1^ at 14 kg N ha^−1^ a^−1^.

**FIGURE 5:**
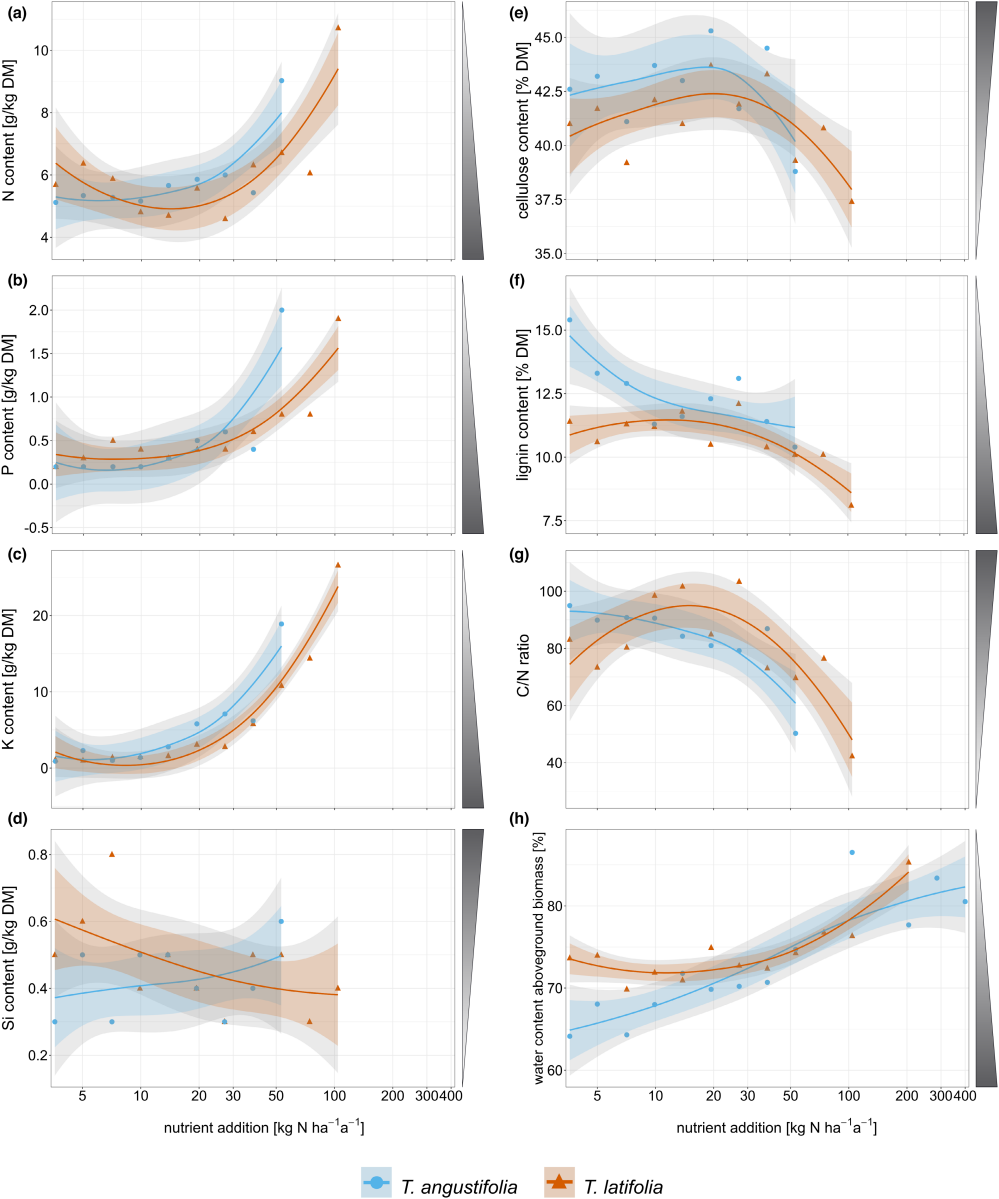
Content [g kg^−1^ dry matter] of (a) N (span = 1.8), (b) P (span = 1.7), (c) K (span = 1.5), and (d) Si (span = 3.0), content [% dry matter] of (e) cellulose (ADF om – ADL, span = 1.4) and (f) lignin (ADL, span = 2.2), (g) mass‐based C/N‐ratio (span = 1.8), and (h) water content [%] (span = 1.3) in aboveground biomass of *Typha angustifolia* (blue) and *Typha latifolia* (red) along the nutrient addition gradient. Dots are original data points, and lines show the smoothed local polynomial regression fitting (loess). 95% confidence intervals are displayed in gray, and 83% confidence intervals are displayed in color. Shaded triangles next to plots indicate preferred (= dark) biomass properties for high‐value utilization. Missing values are due to the fact that sufficient biomass was lacking for the analyses because the plants were growing poorly or had died.

**FIGURE A6:**
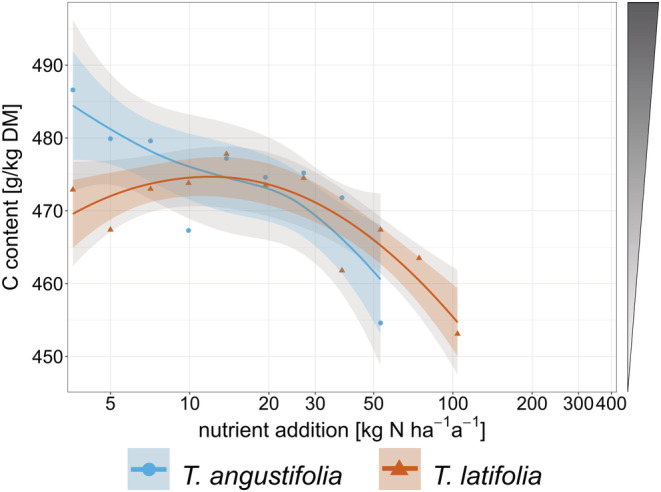
C content [g kg^−1^ dry matter] (span = 1.7) in aboveground biomass of *Typha angustifolia* (blue) and *Typha latifolia* (red) along the nutrient addition gradient. Dots are original data points, and lines show the smoothed local polynomial regression fitting (loess). 95% confidence intervals are displayed in gray, and 83% confidence intervals are displayed in color. The shaded triangle next to the plot indicates preferred (= dark) biomass properties for high‐value utilization. Missing values are due to the fact that sufficient biomass was lacking for the analyses because the plants were growing poorly or had died.

The chemical composition of aboveground biomass showed pronounced effects of the nutrient availabilities but little to no differentiation between the two species (Figure 5). At nutrient additions higher than 53.1 kg N ha^−1^ a^−1^ for *T. angustifolia* and 104.1 kg N ha^−1^ a^−1^ for *T. latifolia*, biomass production was not sufficient for the analysis of nutrient and fiber content because the plants were growing poorly or had died (Figure 5). Macronutrient concentration in aboveground biomass increased significantly with increasing nutrient addition (Figure 5a–c). Accordingly, C content and C/N‐ratio decreased significantly with increasing nutrient addition (Figures A6 and 5g). Si concentrations showed no significant response (Figure 5d).

Nutrient addition had no influence on cellulose content in either species and species did not differ in cellulose content (Figure 5e). Lignin content decreased significantly with increasing nutrient addition in both species (Figure 5f). In *T. angustifolia*, lignin content already decreased from low nutrient addition onwards while in *T. latifolia*, it was stable up to approx 20 kg N ha^−1^ a^−1^, then decreased. At low nutrient addition (approx 3.6–8 kg N ha^−1^ a^−1^), lignin content was significantly higher in *T. angustifolia* than in *T. latifolia*.

The water content of aboveground biomass increased significantly for *T. angustifolia* over the entire nutrient gradient from 65% to 82% (Figure 5h). *T. latifolia* showed an increase from 72% at 11 kg N ha^−1^ a^−1^ to 84% at high nutrient addition.

### 3.3 Practitioner survey

In total, 39 companies were contacted of which five responded with a fully completed questionnaire. All companies made a statement of preferred water content and almost all agreed that little or no other vegetation (or other impurities) should be included in the required biomass (Table 3). Only two companies required certain morphological parameters and two companies a certain chemical composition of the biomass for their products. The other companies did not respond or gave the following reasons for not having filled in the questionnaire: No time for a questionnaire, trade or sale of products only (no production), and no statements about biomass requirements can be made.

**TABLE 3:** Results of the practitioner survey. Five companies responded with a fully completed questionnaire regarding currently used plant biomass, products, and biomass quality requirements

	Current production		Biomass quality requirements			
	Material to be replaced by *Typha* spp.	Products	Water content	Morphological parameters	Chemical composition	Other criteria
1	Sugar cane bagasse; wood cellulose	Disposable tableware (pulp molding)	<10%	‐	Low mineral content	Storable material, free from impurities and other vegetation
2	Perennial grass species	Insulation boards	9%–13% (plant fiber)	Particular fiber size	‐	‐
3	Common reed; reed canary grass	Growing media, substrate	Dry and wet biomass in mixture (e.g., 2:1)	‐	‐	Whole plant used, no impurities (rocks, plastic, ropes)
4	Stalk biomass (hemp)	Insulation material, lightweight construction material, etc.	10–15%	Minimum diameter 2 × 2.5 cm (elliptical shoots); plants as large as possible	‐	Only small amount of impurities, small amount of other vegetation
5	Common reed	CO_2_‐neutral charcoal	<30%	‐	High carbon content, low amount of harmful substances	Pure biomass of one species, no other vegetation, or consistent species composition ratio

## 4 DISCUSSION

The concept of paludiculture has been proposed as a sustainable land‐use option for peatlands for a long time already (Wichtmann & Joosten, 2007), yet large‐scale practical implementation is still at the beginning. The reasons for this are manifold, including the political framework, economic uncertainties but also lack of knowledge on the selection of suitable species or varieties for given environmental conditions. Our aim in this study was to investigate under which environmental conditions the *Typha* species *T. latifolia* and *T. angustifolia* perform best regarding their aboveground and belowground productivity and aboveground biomass quality for high‐value utilization to provide references for practitioners to select the appropriate species for specific field conditions and intended use.

### 4.1 Performance of *Typha* spp. in the water table gradient

The optimum of biomass production was, contrary to hypothesis 1a, not at water tables close to soil surface. Productivity of *T. angustifolia* remained stable over the whole water table gradient and productivity of *T. latifolia* was even higher under dry than under wet conditions. The same pattern was found for photosynthetic rate: It declined with increasing water table in *T. latifolia*, in line with the second part of hypothesis 1a but was stable in *T. angustifolia*. Both species coped well with the driest conditions in our experiment (water table: −45 cm). Similarly, Giannini et al. (2019) reported that a shallow water depth favored the growth of *T. latifolia* compared to deeper water depths. On the other hand, our results are in contrast to several studies reporting that *Typha* is negatively affected by drought (da Cunha Cruz et al., 2019; Geurts & Fritz, 2018; Li et al., 2004) due to its shallow root system being restricted mainly to the upper 15–30 cm of the soil (Geurts & Fritz, 2018). The fact that plants still received rainfall and bi‐weekly fertilizer solution might have reduced the water stress in our experiment by elevating soil moisture in the mesocosms. Water tables, however, fluctuated only within 1–2 cm even after the strongest rainfall events. Macronutrient concentrations in plant biomass, as a measure of plant available nutrients (Ławniczak, 2011), did not correlate with productivity in either species, indicating that higher nutrient concentrations in the soil water of dry compared to flooded mesocosms (Table A1) did not drive the observed pattern in productivity.

In our study, water table did not affect the performance of *T. angustifolia*, but that of *T. latifolia*, whose productivity and photosynthetic rate decreased significantly with increasing water table. Flooding (approx. from +20 cm), however, led to an increase in *T. angustifolia* plant height, which has been shown for several *Typha* species (Grace, 1989; Grace & Wetzel, 1982; Heinz, 2012). Although we did not see a clear optimum in performance of *T. angustifolia* at high water tables, the species seems to cope better with flooding than *T. latifolia*, a fact that is supported by previous studies (Grace & Wetzel, 1981). Additionally, it was found that *T. latifolia* could not aerate its substrate significantly (Grace, 1988). In competition, *T. latifolia* can outcompete *T. angustifolia* in shallow water because of a greater leaf surface area, but *T. angustifolia* is well‐adapted to growing in deeper water depths due to its narrow leaves and large rhizome storage (Grace & Wetzel, 1981, 1982). Our hypothesis 3a can only be confirmed in so far as *T. latifolia* showed optimum performance at low water tables and *T. angustifolia* could cope well with high water tables, but we could not confirm different optima of performance along the water table gradient for the two species since *T. angustifolia* did not show an optimum in performance.

### 4.2 Performance of *Typha* spp. in the nutrient addition gradient

Biomass and morphology, as well as photosynthetic rate of both *Typha* species were, contrary to hypothesis 2a, largely unaffected by low to intermediate nutrient addition but declined at high nutrient addition. Likewise, Ren et al. (2019) and Geurts et al. (2020) reported that *T. latifolia* can perform well at low nutrient addition. Our results suggest that this tolerance to low nutrient levels does not only apply to *T. latifolia* but also to *T. angustifolia*. It should be noted that the nutrient addition values in our study only refer to the nutrient addition with the fertilizer solution, excluding additional nutrients from the substrate, irrigation water, or atmospheric deposition. Hence, actual nutrient availability was likely higher than the indicated nutrient addition. Atmospheric deposition is estimated to add approximately 10 kg N ha^−1^ a^−1^ in our study area (Erisman et al., 2015), roughly doubling the lowest nutrient addition level over the course of the experiment. To keep the additional nutrient supply by the substrate as low as possible, we used nutrient‐poor bog peat instead of fen peat.

Performance of *T. latifolia* and *T. angustifolia* had different optima along the nutrient addition gradient, contradicting hypothesis 3b. Optima of productivity occurred at lower nutrient concentrations in *T. angustifolia* than in *T. latifolia*. This could be related to the fact that the performance of *T. angustifolia* remains stable at low nutrient addition while performance of *T. latifolia* shows a nonsignificant trend to decrease. It could also be that *T. latifolia* has a higher tolerance to stress at high nutrient addition than *T. angustifolia*.

In contrast to the decrease in plant performance observed in both *Typha* species at high nutrient addition in our study, biomass production and photosynthetic rate are often found to be enhanced by an increased nutrient availability even beyond the levels applied in our study (Deegan et al., 2012; Geurts & Fritz, 2018; Grace, 1988; Jordan et al., 1990; Macek & Rejmánková, 2007; Miao et al., 2000; Ren et al., 2019; Santos et al., 2015; Svengsouk & Mitsch, 2001; Wetzel & van der Valk, 1998). Previous studies have shown that high nutrient concentrations (>100 g L^−1^ ammonia) can be harmful to *Typha* and induce morphological and physiological changes in the plants (Chikov et al., 2012; Clarke & Baldwin, 2002; Hong et al., 2020; Jinadasa et al., 2008; Li et al., 2011). In a study with maize, Magalhães et al. (1995) found that plant growth was lower with ${\mathrm{NH}}_{4}^{+}$‐ than with ${\mathrm{NO}}_{3}^{-}$ fertilization under low oxygen supply. In the ${\mathrm{NH}}_{4}^{+}$ fertilized plants they observed higher levels of free NH_3_, which was negatively correlated with growth. It is known that NH_3_ is toxic to plants (Vines & Wedding, 1960). The equilibrium between ${\mathrm{NH}}_{4}^{+}$ and NH_3_ depends on pH and temperature. At a temperature of 30°C and pH of 9.5, as measured in our fertilizer solution, it is heavily shifted towards NH_3_ (Kadlec & Wallace, 2009). Considering this fact, and the anoxic conditions in the mesocosms, the unpleasant smell of the fertilizer solution and unspecific symptoms of plants, yellow leaves, becoming soft and dying quickly lead us to suspect that the fertilizer solution had an elevated NH_3_ content, which led to NH_3_ poisoning of the plants in the levels with high nutrient addition. It seems unlikely that such elevated NH_3_ concentrations occur under field conditions and should be kept in mind when interpreting the performance of *Typha* in this part of the gradient.

### 4.3 *T. latifolia* outperforms *T. angustifolia*

In contrast to hypothesis 3c, *T. latifolia* outperformed *T. angustifolia* regarding productivity, growth, and photosynthetic rate throughout the range of nutrient availability and water table depths of our experiments. This is in contrast to North American studies, which report *T. angustifolia* to be more productive than *T. latifolia* under experimental (Grace & Wetzel, 1981) and field conditions (Dubbe et al., 1988).

Under field conditions, *T. angustifolia* can develop denser stands with more shoots per area (Pfadenhauer & Wild, 2001), which could account for the higher yield compared with *T. latifolia* in the studies mentioned. Due to limited space in our mesocosms, rhizomes could not spread naturally but grew curled up inside the plastic tubes, which may have affected the observed shoot density. It is also possible that stand age plays a role (Geurts et al., 2020; Tanaka et al., 2004) or that *Typha* genotypes differ inherently, as is known from *Phragmites australis* (Achenbach et al., 2012).

Despite young plant age, we observed yields of 9.1–20.4 t dm ha^−1^ a^−1^ for *T. latifolia* and 2.3–11.9 t dm ha^−1^ a^−1^ for *T. angustifolia* in the water table gradient and of 0.4–16.7 t dm ha^−1^ a^−1^ for *T. latifolia* and 0.2–10.9 t dm ha^−1^ a^−1^ for *T. angustifolia* in the nutrient addition gradient. These yields are in the same order of magnitude as in field studies and maximum yields are relatively high compared with studies in Central Europe (Geurts et al., 2020; Maddison, Soosaar, et al., 2009; Zerbe et al., 2013) possibly due to favorable conditions in the mesocosms.

### 4.4 Preferable conditions vary depending on intended biomass use

The biomass requirements of *Typha* species for utilization options have to be identified to enable farmers to produce biomass of suitable quality. However, only few requirements are established. We conducted a practitioner survey to gain more insights into quality requirements to better link research and practice. While the participants could give clear information on preferred water content and morphology, notably there were less clear requirements for element content that were not quantified.

In the following, we attempt to evaluate the results from our experiments for potential utilizations based on the results of the practitioner survey and findings from the literature.

Some building materials and their production processes require, among other criteria, specific morphological parameters such as large plants and a certain shoot diameter (>4 cm diameter for the TYPHABOARD, (Georgiev et al., 2019; Krus et al., 2014; Theuerkorn, 2022), >2 × 2.5 cm diameter of elliptical shoots for building material (survey, Table 3)). In our experiments, both species did not reach these targets, probably due to their age (first growing season) and spatial limitation in the tubes. Under field conditions, *T. latifolia* can reach a diameter of 10 cm (2^nd^ growing season, unpublished own data) and *T. angustifolia* can reach a diameter of 5 cm (Asaeda et al., 2005). In our study, *T. latifolia* had a greater diameter than *T. angustifolia* under most conditions and would therefore be the preferred species for the above‐mentioned building materials, based on diameter. Water level and nutrient addition did not influence culm diameter in *T. latifolia*, while the maximum culm diameter of *T. angustifolia* decreased significantly with increasing nutrient addition. This is in contrast to other studies such as Ren et al. (2019), where, among other traits, maximum shoot diameter increased with increasing nutrient availability (up to 300 kg N ha^−1^ a^−1^) in *T. latifolia*. Plants not fulfilling diameter requirements for building material and insulation boards can still be used for products that do not require specific morphological properties, e.g., blow‐in insulation.

Chemical composition of the biomass can play a role in different utilization pathways: For processing into disposable tableware, a low mineral content of raw biomass can be beneficial (Table 3). For processing into charcoal, a high C content is desirable (Table 3). For bio‐based building material, the content of macronutrients N, P, and K in the biomass seems to have no relevant influence, as long as it is used in dry surroundings, e.g., as indoor insulation material. In a humid environment, however, e.g., due to leakage and inflow of humid air (Krus et al., 2014), a low nutrient content in the biomass can possibly be desirable for lower degradability (e.g., if the material is not treated with chemicals), as is known for common reed as thatching material (Greef & Horlings, 2016). Higher biomass C/N‐rates can further be beneficial for durability, as they are negatively correlated with decomposition (Chimney & Pietro, 2006). Additionally, a high Si content is favorable as it leads to building material that is more durable, resistant to decay, less flammable, and insulates better against heat and cold (Vasanthi et al., 2012). In the water table gradient, the two species showed different patterns of biomass nutrient content. While nutrient content of *T. latifolia* stayed in a narrow range over the gradient, nutrient content of *T. angustifolia* decreased significantly at high water tables. Biomass nutrient content increased for both species with increasing nutrient addition. In both species, Si content did not change over the nutrient addition gradient but increased 2‐ to 3‐fold at high water tables. We assume that high water tables lead to increased uptake and storage of Si in the plant tissue of *Typha* to increase the plants' stability.

Assuming that a low content of N, P, K, a high content of C, Si, and high a C/N‐ratio are beneficial, we conclude that biomass quality in terms of element content decreases with increasing nutrient availability and, in particular for *T. angustifolia*, increases with increasing water table. The trade‐off between increasing productivity but decreasing biomass quality with increasing nutrient addition emphasizes the importance of adapting the utilization depending on site nutrient availability.

A low water content in aboveground biomass is desirable for storage and processing into various products (Table 3). To reduce drying time and effort, a low initial water content is preferable. At water tables between 0 and +20 cm, the water content of aboveground biomass harvested in our experiment matches findings from field harvests in late autumn (Maddison, Mauring, et al., 2009). Lowest water content was reached at low water tables and low nutrient addition in our experiment, but even the lowest water content was far above 20%, so active drying of the biomass is necessary for most applications. Again, the trade‐off between high productivity and short drying time requires prioritization of one over the other regarding desired growing conditions.

For paper and new utilization options like biodegradable packaging, a high cellulose content close to 40% and low lignin content below 30% are required (Ververis et al., 2004). We found *Typha* biomass to be suitable for paper production: In our study, both species contained 35%–50% cellulose and 10%–13% lignin. *T. angustifolia* had significantly higher cellulose content at high water tables, whereas the water table had no influence on lignin content in either species. Along the nutrient addition gradient, cellulose content was optimal at medium nutrient addition (approx. 20 kg N ha^−1^ a^−1^) while lignin content decreased with increasing nutrient addition. If packaging is to be used in the food sector, the absence of harmful substances such as herbicides and heavy metals is another requirement. Here, we did not test for herbicides and heavy metals as their occurrence in our mesocosm setting, far from agricultural landscapes, would not be transferable to field conditions. Such substances since the mesocosms were closed systems without contact with surface water or agricultural fields that would have exposed them to herbicides or heavy metals.

In addition to the factors investigated in this study, water table and nutrient supply, there are other factors that can strongly influence yield, tissue chemical composition, and water content of *Typha* biomass in field cultivation, among them are harvest date, stand age, water level fluctuations, interspecific competition and genotype (Geurts et al., 2020; Kasak et al., 2020; Maddison, Mauring, et al., 2009; McNaughton, 1974; Tanaka et al., 2004). For high‐value utilization, which was the focus of this study, biomass is preferably harvested in winter (Wichtmann, 2016). However, aboveground biomass harvested in winter reaches a lower yield and generally has lower nutrient concentrations, lower fiber, and higher protein content, than summer harvested biomass, as biomass allocation and nutrients are shifted to belowground organs towards winter (Dubbe et al., 1988; Kasak et al., 2020; Maddison, Mauring, et al., 2009; Pijlman et al., 2019). Furthermore, in the first years after planting, the productivity of *Typha* stands increased with increasing stand age and, in other paludicrops, ash content increased while the content of elements relevant for combustion decreased (Garver et al., 1988; Giannini et al., 2016).

### 4.5 Insights for paludiculture

In this study, we found that *Typha* productivity was surprisingly tolerant of low nutrient supply and its biomass quality even benefitted under such conditions. Low nutrient levels are to be expected at established paludiculture sites after several seasons with a yearly nutrient removal by harvest (Geurts et al., 2020; Hinzke et al., 2021). As the levels with low nutrient addition in this study mimic such a nutrient‐poor field site and the *Typha* plants reached comparable aboveground productivity to established stands, despite their young age and possibly lower nutrient storage capacity in the rhizome, we conclude that it is possible to maintain an unfertilised paludiculture in the long term and thus counteract eutrophication of the environment. In paludiculture, rewetting is primarily aimed at providing urgently needed climate benefits in the form of C sequestration, reducing greenhouse gas emissions, and providing other ecosystem services such as water storage and improvement of water quality by removal of nutrients and pollutants (Bhatia & Goyal, 2014; Biancalani et al., 2014; Dion & McCandless, 2013; Grosshans, 2014). This can only be achieved by keeping the water table at soil surface and above, which is a prerequisite for paludiculture. Due to small‐scale soil unevenness, rewetting of formerly drained peatlands will result in a mosaic of different water table depths. In a large‐scale field cultivation, however, it is not possible to make such small‐scale differences in crop cultivation or utilization to ensure optimal cultivation everywhere. Therefore, species that provide a consistent quality over a wide range of water table depths are desirable. Our finding that biomass yield of *T. angustifolia* and tissue chemical composition of *T. latifolia* are largely unaffected by water table depth are therefore promising results. Low water tables were beneficial for *T. latifolia* productivity, while high water tables were beneficial for *T. angustifolia* biomass quality.

With regard to utilization, our results imply that when plant size (diameter) or biomass yield is important, e.g., for certain building materials, *T. latifolia* is generally preferable over *T. angustifolia*. When a low content of macronutrients (N, P, K), high content of C, Si, and high C/N‐ratio are beneficial, both species should be cultivated at low nutrient supply and *T. angustifolia* is preferable at high water tables. When cellulose and lignin content are important, e.g., for paper and biodegradable packaging, both species should be cultivated at medium nutrient supply (~20 kg N ha^−1^ a^−1^) and *T. angustifolia* is again preferable at high water tables.

## AUTHOR CONTRIBUTIONS

**Kerstin Haldan:** involved in conceptualization (equal), formal analysis (lead), investigation (equal), methodology (equal), supervision (equal), visualization (lead), writing—original draft (lead), and writing—review and editing (equal). **Nora Köhn:** involved in formal analysis (supporting), investigation (equal), visualization (supporting), writing—original draft (supporting), and writing—review and editing (equal). **Anja Hornig:** involved in investigation (supporting) and writing—review and editing (supporting). **Sabine Wichmann:** involved in conceptualization (supporting), funding acquisition (equal), project administration (lead), and writing—review and editing (equal). **Juergen Kreyling:** involved in conceptualization (equal), funding acquisition (equal), methodology (equal), project administration (supporting), supervision (equal), and writing—review and editing (equal).

## CONFLICT OF INTEREST

The authors have no conflict of interest to declare.

## Data Availability

The data used in this study for direct analysis and visualization are available in Figshare, DOI: 10.6084/m9.figshare.20278650.

## APPENDIX A

**TABLE A1:** Results of soil water analysis

	Total N [mg L^−1^]	Ammonium [mg L^−1^]	Nitrate [mg L^−1^]	Ortho‐phosphate [mg L^−1^]	Potassium [mg L^−1^]
Method	DIN EN ISO 11905‐1	DIN EN ISO 11732	DIN EN ISO 10304‐1	DIN EN ISO 15681‐1	DIN EN ISO 11885
Water table [cm]					
−45	7.1	<0.04	8.5	15	59.3
−38	25	<0.04	90	14	64.3
−31	14	<0.04	40	1.4	33.1
−24	39	<0.04	130	4.8	67.7
−18	26	<0.04	80	5.3	55.1
−12	3.2	<0.04	0.99	0.88	31.9
−6	4.3	<0.04	2.1	0.44	31.5
0	4.2	<0.04	2.0	2.6	65.4
+5	1.6	<0.04	<0.10	1.9	3.4
+10	1.7	<0.04	<0.10	0.95	4.1
+16	1.3	<0.04	<0.10	0.10	1.8
+22	1.2	<0.04	<0.10	0.18	2.4
+28	0.86	<0.04	<0.10	0.060	<1.0
+34	1.3	<0.04	<0.10	0.33	1.7
+40	1.0	<0.04	<0.10	0.21	2.7
Nutrient addition [kg N ha^−1^ a^−1^]					
3.6	4.4	<0.04	2.5	0.038	<1.0
5.0	3.9	<0.04	<0.10	<0.030	<1.0
7.1	3.6	<0.04	0.53	0.038	<1.0
9.9	3.8	<0.04	0.19	0.40	1.9
13.8	3.0	<0.04	0.25	0.24	1.4
19.4	3.6	0.06	0.72	1.00	7.4
27.1	3.5	<0.04	0.37	0.065	7.2
37.9	4.6	<0.04	2.3	0.26	8.0
53.1	5.9	0.66	11	2.6	60.5
74.4	15	11	26	18	124
104.1	23	9.4	56	38	292
145.8	88	49	240	76	450
204.1	79	57	150	66	798
285.7	230	97	560	140	1220
400.0	720	300	1300	330	1860

*Note*: Soil water was extracted on 8 September 2019 (nutrient addition gradient) and 12 September 2019 (water table gradient) 1 week after the last nutrient addition treatment. Soil water samples were collected from a depth of 5–15 cm below the soil surface in the planted plastic tubes using Rhizon MOM soil moisture samplers (Rhizosphere research products, Wageningen, The Netherlands (Meijboom & van Noordwijk, 1991)). Samples were analyzed by Industrie‐ und Umweltlaboratorium Vorpommern GmbH, Greifswald.
